# Eating order and childhood obesity among preschoolers in China: A cross-sectional study

**DOI:** 10.3389/fped.2023.1139743

**Published:** 2023-03-08

**Authors:** Jin Dai, Jingyun Yang, Hailing Fan, Yixin Wu, Huilan Wu, Yun Wang, Tao-Hsin Tung, Lizhen Wang, Meixian Zhang

**Affiliations:** ^1^ Department of Pediatrics, Taizhou Hospital of Zhejiang Province Affiliated to Wenzhou Medical University, Taizhou Enze Medical Center (Group) Enze Hospital, Taizhou, China; ^2^ Department of Pediatrics, Taizhou Hospital of Zhejiang Province Affiliated to Wenzhou Medical University, Linhai, China; ^3^ Evidence-Based Medicine Center, Taizhou Hospital of Zhejiang Province Affiliated to Wenzhou Medical University, Linhai, China

**Keywords:** eating order, obesity, overweight, child, preschool, vegetables

## Abstract

**Background:**

Early childhood is a critical period for dietary education and development of good eating habits. However, few studies have investigated the effect of eating order in children and childhood obesity in real-world settings.

**Objective:**

To examine whether the order in which meats/fish or vegetables are consumed affects the risk of obesity in preschoolers.

**Methods:**

We conducted a population-based cross-sectional study using a self-administered online survey on the lifestyle and health behaviors of preschoolers in Taizhou, China. A total of 3,200 parents were invited to take part in the survey, and 2,049 of them completed the questionnaire. Children were classified as having a normal weight, overweight, or obesity using the definitions provided by the International Obesity Task Force, and z-scores for body mass index were calculated. We divided the children's eating order at the beginning of the meal into two groups: “vegetables before meats/fish” or “meats/fish before vegetables”. We analyzed the relationship between what was consumed first at a meal and the overweight status of each child.

**Results:**

No difference in body mass index was observed between the children eating meats/fish-first and the children eating vegetables-first during a meal. Children with parents who were affected by obesity were more likely to eat vegetables first. Among children of mothers with obesity, body mass index was significantly higher in the meats/fish-first group than that in the vegetable-first group (2.891 vs. 0.845, *P* = 0.007). In children whose mothers were affected by obesity, those that ate meats/fish first had a 12.21 times higher risk of being overweight compared with those that ate vegetables first (95% CI:1.22–121.74, *P* = 0.033).

**Conclusion:**

Our findings suggest eating vegetables or meats/fish at the start of a meal does not affect weight status in preschoolers.

## 1. Introduction

Childhood obesity remains a global public health challenge. An estimated 39 million children under the age of 5 years were affected by overweight or obesity in 2020 (1). In China, childhood obesity has increased rapidly over the past four decades, with the latest prevalence being 6.8% for overweight and 3.6% for obesity in children younger than 6 years old, and 11.1% for overweight and 7.9% for obesity in children and adolescents aged 6–17 years (2). Overweight and obesity affect children's physical and mental health, leading to a series of adverse health outcomes such as cardiovascular disease, type 2 diabetes, and cancer (3). Additionally, school children with overweight or obesity are more vulnerable to bullying and unfair treatment while in school, which can impede their healthy development.

It is well known that the early prevention of obesity is much better than the treatment of obesity at later stages (4). Several preventable risk factors for childhood overweight have been identified, however there is still a lack of effective long-term interventions (5, 6). Poor dietary habits and low consumption of healthy foods are risk factors for mortality, whereas good eating habits are vital to a lifetime of health and can help prevent obesity. A study of the general population in Japan showed that modifying unhealthy eating habits and avoiding the accumulation of multiple unhealthy eating habits is important for reducing the risk of obesity (7). The order in which food is eaten at the start of a meal varies among individuals. The traditional Chinese diet consists of mainly grains, vegetables, poultry, pork and fish (8). We usually serve cold dishes, drinks and wine first, followed by hot dishes (vegetables, meat, fish or soup), followed by the staple food (rice or wheat), and finally the desert and fruits. In a family, we usually eat together after serving the staple food. Interestingly, eating order was also indicated to be associated with the risk of childhood obesity (9), as consuming vegetables first at a meal may not only reduce meal energy intake, but also increase vegetable intake (10). A survey of preschool children in Changsha, Hunan Province found that daily consumption of vegetables was reported by 64.4% of children (11). However, few studies have investigated the relationship between the eating order of meats/fish and vegetables, and childhood obesity. Given the low levels of vegetable intake and the increasing obesity epidemic, we hypothesized that the eating order of meats and/or fish and vegetables during a meal may be associated with childhood overweight and obesity in China.

Dietary patterns established in early childhood can persist into adulthood (9, 12). The pattern and quality of diet in childhood are longitudinally associated with weight and cardiometabolic markers (13, 14). Therefore, early childhood is a critical period for dietary education and development of eating habits (15). Our study aims to provide evidence for kindergarten teachers and parents to carry out dietary education for young children. To achieve this, we conducted a survey to investigate the eating habits of preschoolers and to explore the relationship between the eating order of meat and vegetables and childhood obesity.

## 2. Methods

### 2.1. Study design and participants

Based on a cross-sectional design, we conducted a convenience sampling to recruit eight kindergartens in Taizhou, which is a city with unique cultural and economic background, located in the middle coast of Zhejiang Province, China. A digital survey was delivered to the parents of preschoolers by their teachers using the WeChat-combined Wen-Juan-Xing platform, which was a platform that distributes self-administered questionnaires electronically to others. The participants voluntarily answered the self-administered survey by scanning the Quick Response code on their mobile phones from December 1, 2021, to January 31, 2022. A total of 3,200 parents were invited to take part in the survey,and 2,049 of them completed the questionnaire with a response rate of 64.03%. The exclusion criteria included duplicate submissions (*n* = 10), children younger than 3 years of age or older than 7 years (*n* = 29), and extreme height or weight values defined by outside mean ± 3 SD (*n* = 75). This study focused on the effect of eating order on overweight and obesity rather than thinness, so children with thinness (*n* = 419) were also excluded according to the International Obesity Task Force (IOTF) BMI cutoffs. After quality control, 1,562 interviewees with valid data were included in the study.

The purpose of the survey are explained in the introduction section. Voluntary participation in the survey was considered as the receipt of informed consent from both the children and their parents. This study was approved by the ethics committee of the Taizhou Hospital of Zhejiang Province (approval no.: K20220123) in China. All procedures were performed in compliance with the guidelines of our institutional ethics committee and the tenets of the 1975 Declaration of Helsinki. All information of the respondents was kept anonymous.

### 2.2. Children's and parental weight status

The interviewees reported data on the height and weight of their child and of the child's parent(s). Body mass index (BMI) was calculated as the weight (in kilograms) divided by the square of height (in meters). Based on the International Obesity Task Force (IOTF), each child's BMI was adjusted for age and sex, and converted to a standardized z-score. Children were categorized as having a normal weight, overweight, or obesity (16). Parental weight status was classifi−ed as having a underweight (BMI < 18.5 kg/m^2^), normal weight (18.5 kg/m^2^ ≤ BMI < 25 kg/m^2^), and overweight/obesity (BMI ≥ 30 kg/m^2^), based on international thresholds (17).

### 2.3. Order of eating

The eatin−g order exhibited by children was assessed by the following question: “Does your child usually eat vegetables or meats/fish first at start of meal?” The following two response options were provided: “vegetables before meats/fish” or “meats/fish before vegetables”.

### 2.4. Covariates

The questionnaire collected sociodemographic information on the sex, date of birth, residence (urban and rural), and parental education level of each child. The one-child family was inferred by the following question, “How many siblings does the child have?” (0, 1, or ≥2). Annual household income was measured by the following question, “What was your total household income last year?” (<120,000, 120,000–500,000, or >500,000 Chinese Yuan [CNY]).

### 2.5. Statistical analysis

Categorical variables, including demographic, parental, and familial characteristics, were expressed as counts and percentages. Chi-squared tests were used to compare differences in these characteristics between the vegetables-first group and meats/fish-first group. The difference in BMI z-scores in children with different eating orders was tested using the Student's t-test. Odds ratios (OR) and 95% confidence intervals (CI) were calculated to assess the association between what was consumed first at a meal and the overweight status of each child using a logistic regression model. Place of residence, one-child family, income, and parental educational levels were adjusted as covariates. Statistical analyses were performed using the IBM SPSS software (version 26.0, SPSS Inc.). The tests were two-sided with the significance set at *P* ≤ 0.05.

## 3. Results

A total of 1,562 children aged 3–6 years with a mean BMI z-score of 0.725 ± 1.600 were analyzed in this study, of which 830 were boys and 732 were girls. More than half of the children had a normal weight, while the proportion of overweight and obesity were 10.3% and 12.6%, respectively. Overall, the proportion of vegetables and meats/fish consumed first at the start of the meal were 52.1% and 47.8%, respectively. The sociodemographic characteristics of the study participants are summarized in Table 1. Compared with children who ate vegetables first, the children who ate meats/fish first had higher levels of parental education and annual household income, a higher proportion of urban residents and one-child families, and a higher proportion of fathers who were affected by overweight and obesity (*P* < 0.05).

**Table 1:** Basic characteristics of participants (*N* = 1562).

Variables	Total (n = 1562)	Food consumed first		*P*
		Vegetables (n = 814, 52.1%)	Meat/Fish (n = 748, 47.9%)	* *
***Child-related characteristics***				
BMI Z score	0.725 ± 1.600	0.768 ± 1.638	0.679 ± 1.554	0.274
Body weight status				0.438
Normal weight	1,204 (77.1)	619 (76.0)	585 (78.2)	
Overweight	161 (10.3)	84 (10.3)	77 (10.3)	
Obesity	197 (12.6)	111 (13.6)	86 (11.5)	
Sex				0.386
Boy	830 (53.1)	424 (52.1)	406 (54.3)	
Girl	732 (46.9)	390 (47.9)	342 (45.7)	
Age (years)				0.159
3	375 (24.0)	177 (21.7)	198 (26.5)	
4	500 (32.0)	266 (32.7)	234 (31.3)	
5	533 (34.1)	291 (35.7)	242 (32.4)	
6	154 (9.9)	80 (9.8)	74 (9.9)	
***Parental-related characteristics***				
Father's age	37.1 ± 11.1	37.5 ± 14.2	36.6 ± 6.0	0.109
Mother's age	35.2 ± 6.8	35.4 ± 7.8	34.9 ± 5.4	0.095
Father's education level				**<0.001**
Junior or below	233 (14.9)	140 (17.2)	93 (12.4)	
Senior	433 (27.7)	254 (31.2)	179 (23.9)	
University or above	896 (57.4)	420 (51.6)	476 (63.6)	
Mother's education level				**<0.001**
Junior or below	220 (14.1)	144 (17.7)	76 (10.2)	
Senior	324 (20.7)	194 (23.8)	130 (17.4)	
University or above	1,018 (65.2)	476 (58.5)	542 (72.5)	
Father's BMI category				**0.005**
Normal weight	854 (57.7)	466 (61.6)	388 (53.7)	
Overweight	541 (36.6)	247 (32.6)	294 (40.7)	
Obesity	84 (5.7)	44 (5.8)	40 (5.5)	
Mother's BMI category				0.527
Normal weight	1,304 (87.5)	663 (86.6)	641 (88.5)	
Overweight	152 (10.2)	84 (11.0)	68 (9.4)	
Obesity	34 (2.3)	19 (2.5)	15 (2.1)	
***Family-related characteristics***				
Residence				**<0.001**
Urban	1,011 (64.7)	483 (59.3)	528 (70.6)	
Rural	551 (35.3)	331 (40.7)	220 (29.4)	
One-child family				**0.021**
Yes	669 (42.8)	326 (40.0)	343 (45.9)	
No	893 (57.2)	488 (60.0)	405 (54.1)	
Annual household income (CNY)				**0.006**
<120,000	330 (21.1)	197 (24.2)	133 (17.8)	
120,000–500,000	1,000 (64.0)	506 (62.2)	494 (66.0)	
>500,000	232 (14.9)	111 (13.6)	121 (16.2)	

Table 2 presents the prevalence of overweight and obesity among children with different parental weight statuses. Children with parents who were affected by obesity had higher percentages of overweight and obesity, regardless of whether the father or mother had obesity (*P* < 0.05).

**Table 2:** Prevalence of overweight and obesity among children with different parental weight status.

Parental weight status	Child's weight status			*P*
	Normal	Overweight	Obesity	
Mother's BMI groups				**<0.001**
Non-obesity (*n* = 1456)	1,141 (78.4)	147 (10.1)	168 (11.5)	
Obesity (*n* = 34)	16 (47.1)	6 (17.6)	12 (35.3)	
Total (*n* = 1490)	1,157 (77.7)	153 (10.3)	180 (12.1)	
Father's BMI groups				**0.007**
Non-obesity (*n* = 1395)	1,103 (79.1)	134 (9.6)	158 (11.3)	
Obesity (*n* = 84)	54 (64.3)	15 (17.9)	15 (17.9)	
Total (*n* = 1479)	1,157 (78.2)	149 (10.1)	173 (11.7)	

We compared the BMI z-scores of children who ate vegetables first and those who ate meats/fish first, stratified by whether their fathers or mothers were affected by obesity. The results are presented in Table 3. Only in children whose mother was affected by obesity, the BMI z-score of those who ate vegetables first (*n* = 19) was significantly lower than that of those who ate meats/fish first (*n* = 15) (0.845 ± 1.984 vs. 2.891 ± 2.098, *P* = 0.007).

**Table 3:** Differences in BMI z-score between children who consumed vegetables first and those consumed meat/fish first at a meal, stratified by parental weight status.

Stratification	*n_1_*/*n_2_*	Food consumed first		*P*
		Vegetables	Meat/fish	
Mother's BMI groups (*n* = 1490)	766/724			
Non-overweight	663/641	0.684 ± 1.579	0.585 ± 1.464	0.242
Overweight	84/68	0.900 ± 1.485	1.030 ± 1.807	0.628
Obesity	19/15	0.845 ± 1.984	2.891 ± 2.098	**0.007**
Father's BMI groups (*n* = 1479)	757/722			
Non-overweight	466/388	0.599 ± 1.56	0.579 ± 1.533	0.846
Overweight	247/294	0.768 ± 1.457	0.755 ± 1.533	0.917
Obesity	44/40	1.357 ± 1.823	0.744 ± 1.374	0.088

Table 4 summarizes the effect of eating meats/fish first on the risk of overweight or obesity in preschoolers, stratified by parental weight status. Compared with eating vegetables first, eating meats/fish first was significantly associated with a 12.21 times higher risk of being overweight (95% CI: 1.22–121.74, *P* = 0.033) in children whose mothers were affected by obesity, after adjusting for place of residence, number of children, income, and parental education levels.

**Table 4:** The effect of eating meat/fish first on risk of overweight/obesity in pre-schoolers stratified by parental weight status.

Stratification	*P*	*OR*	95% *CI*
Mother's weight status			
Non-obesity	0.764	0.96	0.75–1.24
Obesity	**0.033**	12.21	1.22–121.74
Father's weight status			
Non-obesity	0.676	1.06	0.81–1.38
Obesity	0.317	0.54	0.16–1.82

Eating vegetables first as reference.

Adjusted for residence, one-child family, income, father's and mother's educational levels.

## 4. Discussion

### 4.1. Familial clustering of obesity

Genetic, environmental, and behavioral factors have been shown to contribute to childhood obesity. Familial clustering of obesity has been reported in several countries (18, 19). Family environmental factors, such as dietary behavior, are important risk factors for increasing obesity in children (20). In a childhood obesity study from Brazil, parents with obesity were emphasized as an independent predictor of childhood overweight (21). Consistent with previous studies, our study also found that parents with obesity had children who were more likely to have obesity. In addition, we found that parents with obesity may have an impact on the order of foods in which their children ate. These findings emphasize the role of family in preventing obesity.

### 4.2. Association of eating order with obesity

Eating order is a specific detail of one's eating habit and often varies from person to person. Previous studies on eating orders have focused on glycemic control in adults with diabetes (22, 23). Few studies have examined the link between eating order and risk of obesity. In our study, we investigated the relationship between the consumption order of meats and vegetables and weight in children through a self-administered survey. We found no significant difference in either BMI z-score or proportion of obesity status between children who ate vegetables-first or meats/fish-first during a meal. However, in a small subsample of children whose mothers were affected by obesity, we found that children who ate meats/fish first were more likely to have obesity than children who ate vegetables first. In a study with 4,040 first-grade students from Tokyo, Japan, it was reported that children who ate meats/fish at the beginning of a meal had a higher risk of being overweight than those who ate vegetables-first (24). There are several possible explanations for the conflicting results. First, there are differences in both genetic and sociodemographic characteristics, including age, family, and socioeconomic status of each study population (25). Second, significant differences in dietary structure and cultures between Chinese and Japanese populations may be responsible for these inconsistent findings (26–28). Finally, in contrast to the World Health Organization's (WHO) Child Growth Standards used in the Japanese study, our study utilized the IOTF BMI cut-offs to assess overweight and obesity in children. Therefore, the prevalence rates of childhood obesity calculated under these two criteria are quite different (16).

### 4.3. Advantages of eating vegetables first

The consumption of vegetables first may have greater benefits than other alternatives. A Japanese study showed that children who ate vegetables first had a higher total vegetable intake than those who did not eat vegetables first (29). A study in the United States reported similar results (10). Eating vegetables first led to increased vegetable intake and reduced food energy intake (10). The vegetable intake of children who ate vegetables first was 93% higher than of that in children who do not often or never eat vegetables first (29). In addition to children, eating vegetables also has many advantages for adults. A study found that eating vegetables first, then meat and/or fish, and then carbohydrates can prevent the fluctuation of postprandial blood glucose and insulin levels (23). For patients with type 2 diabetes, eating vegetables can effectively control short- and long-term blood glucose levels (30). Moreover, increasing intake of vegetables and reducing intake of saturated fats in children with a familial history of obesity early on may help prevent the occurrence of type 2 diabetes (31).

### 4.4. Establishing reasonable eating habits

This study included kindergartens in different districts and levels of Taizhou. This study showed that parents with obesity had an impact on the order in which their children ate food and the risk of obesity in their children. It is known that dietary habits develop during childhood (32). Poor diet during childhood may persist into adulthood and increase one's risk of obesity and obesity-related complications such as type 2 diabetes (2, 33). In our study, children of mothers with obesity who eat meats/fish first had a significantly increased risk of being affected by overweight and/or obesity. Therefore, eating vegetables first during a meal may be a better option, especially for children whose mothers were affected by obesity. Our study could direct further research into the impact of eating order in this subpopulation of children with parents with obesity.

### 4.5. Strengths and limitations

Few studies have examined the relationship between children's eating order and health. Japanese scholars first found that eating vegetables before meats/fish could reduce the risk of obesity (21). This study provides new evidence of the association between eating order and childhood obesity in China. Furthermore, the results of this study can provide a reference for kindergarten teachers in China to conduct dietary education for children.

This study had several limitations. First, the participants were recruited from a single city in southern China, which is not representative of the entire population. Second, when stratified by maternal or paternal obesity, the sample size of the maternal obesity group was only 34. The small subsample size may have affected the results of the analysis. Third, after data quality control, the percentage of valid questionnaire was 76.2%. Fourth, data on weight, height, and eating order were recalled by memory and self-reported by the children's parent, therefore limiting the accuracy of the data due to recall bias. Most studies showed that self-reported height was overestimated and weight and BMI were underestimated (34, 35). Fifth, other possible confounding factors, such as eating behavior, nutrition, exercise, and sleep, were not considered. Sixth, we did not provide the consistency and stability of the order in which children ate at each meal. In the future, With the consent of children's parents, we will carry out a one-week investigation and study, in which the children's eating order of meats/fish or vegetables at the start of a meal will be recorded every day. Seventh, additional aspects of eating order that was not addressed in this study. We did not consider the effect of eating other foods first, such as bread/rice, soup or others. Eighth, we used only one question to measure eating vegetables or meats/fish first at the start of a meal, which may introduce social desirability bias. Finally, considering the cross-sectional nature of the study, we could not clarify the causal relationship between eating order and weight status. Future longitudinal studies can be conducted to further validate this relationship.

## 5. Conclusions

Our study demonstrated that parents with obesity increased the risk of obesity in their children. There is no evidence to support the association between the order of meats/fish and vegetable consumption, and overweight and obesity in children. Further studies may be required before making recommendations on eating order.

## Data Availability

The raw data supporting the conclusions of this article will be made available by the authors, without undue reservation.

## References

[1] World Health Organization. Facts and figures on childhood obesity; 2020. < https://www.who.int/end-childhood-obesity/facts/en/>.

[2] Pan XF Wang L Pan A. Epidemiology and determinants of obesity in China. Lancet Diabetes Endocrinol. (2021) 9(6):373–92. 10.1016/S2213-8587(21)00045-0 34022156

[3] Zhi X Kuang XH Liu K Li J. The global burden and temporal trend of cancer attributable to high body mass index: estimates from the global burden of disease study 2019. Front Nutr. (2022) 9:918330. 10.3389/fnut.2022.918330 35958256 PMC9360580

[4] Pandita A Sharma D Pandita D Pawar S Tariq M Kaul A. Childhood obesity: prevention is better than cure. Diabetes Metab Syndr Obes. (2016) 9:83–9. 10.2147/DMSO.S90783 27042133 PMC4801195

[5] Ebbeling CB Pawlak DB Ludwig DS. Childhood obesity: public-health crisis, common sense cure. Lancet. (2002) 360(9331):473–82. 10.1016/S0140-6736(02)09678-2 12241736

[6] Mead E Brown T Rees K Azevedo LB Whittaker V Jones D Diet, physical activity and behavioural interventions for the treatment of overweight or obese children from the age of 6 to 11 years. Cochrane Database Syst Rev. (2017) 6(6):CD012651. 10.1002/14651858.CD012651 28639319 PMC6481885

[7] Ishida Y Yoshida D Honda T Hirakawa Y Shibata M Sakata S Influence of the accumulation of unhealthy eating habits on obesity in a general Japanese population: the hisayama study. Nutrients. (2020) 12(10):3160. 10.3390/nu12103160 33081125 PMC7602721

[8] Zhen S Ma Y Zhao Z Yang X Wen D. Dietary pattern is associated with obesity in Chinese children and adolescents: data from China health and nutrition survey (CHNS). Nutr J. (2018) 17(1):68. 10.1186/s12937-018-0372-8 29996840 PMC6042200

[9] Nicklaus S Remy E. Early origins of overeating: tracking between early food habits and later eating patterns. Curr Obes Rep. (2013) 2(2):179–84. 10.1007/s13679-013-0055-x

[10] Spill MK Birch LL Roe LS Rolls BJ. Serving large portions of vegetable soup at the start of a meal affected children's Energy and vegetable intake. Appetite. (2011) 57(1):213–9. 10.1016/j.appet.2011.04.024 21596073 PMC3140700

[11] Huo J Kuang X Xi Y Xiang C Yong C Liang J Screen time and its association with vegetables, fruits, snacks and sugary sweetened beverages intake among Chinese preschool children in Changsha, hunan province: a cross-sectional study. Nutrients. (2022) 14(19):4086. 10.3390/nu14194086 36235738 PMC9572133

[12] Movassagh EZ Baxter-Jones ADG Kontulainen S Whiting SJ Vatanparast H. Tracking dietary patterns over 20 years from childhood through adolescence into young adulthood: the Saskatchewan pediatric bone mineral accrual study. Nutrients. (2017) 9(9):990. 10.3390/nu9090990 28885565 PMC5622750

[13] Luque V Closa-Monasterolo R Grote V Ambrosini GL Zaragoza-Jordana M Ferré N Dietary patterns acquired in early life are associated with cardiometabolic markers at school age. Clin Nutr. (2021) 40(7):4606–14. 10.1016/j.clnu.2021.06.001 34229265

[14] Sørensen LMN Aamodt G Brantsæter AL Meltzer HM Papadopoulou E. Diet quality of Norwegian children at 3 and 7 years: changes, predictors and longitudinal association with weight. Int J Obes (Lond). (2022) 46(1):10–20. 10.1038/s41366-021-00951-x 34462565

[15] Lioret S Campbell KJ McNaughton SA Cameron AJ Salmon J Abbott G Lifestyle patterns begin in early childhood, persist and are socioeconomically patterned, confirming the importance of early life interventions. Nutrients. (2020) 12(3):724. 10.3390/nu12030724 32182889 PMC7146362

[16] Cole TJ Lobstein T. Extended international (IOTF) body mass index cut-offs for thinness, overweight and obesity. Pediatr Obes. (2012) 7(4):284–94. 10.1111/j.2047-6310.2012.00064.x 22715120

[17] Obesity: preventing and managing the global epidemic. Report of a WHO consultation. World Health Organ Tech Rep Ser. (2000) 894:i–xii. 1-253. https://pubmed.ncbi.nlm.nih.gov/11234459/11234459

[18] Xi B Mi J Duan JL Yan SJ Cheng H Hou DQ Familial clustering of obesity and the role of lifestyle factors among children in Beijing. Zhonghua Yu Fang Yi Xue Za Zhi. (2009) 43(2):122–7. Chinese. 10.3760/cma.j.issn.0253-9624.2009.02.008 19534904

[19] Li Z Luo B Du L Hu H Xie Y. Familial clustering of overweight and obesity among schoolchildren in northern China. Int J Clin Exp Med. (2014) 7(12):5778–83. https://pubmed.ncbi.nlm.nih.gov/25664106/25664106 PMC4307553

[20] Gonzalez-Casanova I Sarmiento OL Pratt M Gazmararian JA Martorell R Cunningham SA Individual, family, and community predictors of overweight and obesity among Colombian children and adolescents. Prev Chronic Dis. (2014) 11:E134. 10.5888/pcd11.140065 25101491 PMC10842398

[21] Oliveira AM Oliveira AC Almeida MS Oliveira N Adan L. Influence of the family nucleus on obesity in children from northeastern Brazil: a cross-sectional study. BMC Public Health. (2007) 7:235. 10.1186/1471-2458-7-235 17822567 PMC2147025

[22] Jardine MA Kahleova H Levin SM Ali Z Trapp CB Barnard ND. Perspective: plant-based eating pattern for type 2 diabetes prevention and treatment: efficacy, mechanisms, and practical considerations. Adv Nutr. (2021) 12(6):2045–55. 10.1093/advances/nmab063 34113961 PMC8634508

[23] Nishino K Sakurai M Takeshita Y Takamura T. Consuming carbohydrates after meat or vegetables lowers postprandial excursions of glucose and insulin in nondiabetic subjects. J Nutr Sci Vitaminol (Tokyo). (2018) 64(5):316–20. 10.3177/jnsv.64.316 30381620

[24] Tani Y Fujiwara T Ochi M Isumi A Kato T. Does eating vegetables at start of meal prevent childhood overweight in Japan? A-CHILD study. Front Pediatr. (2018) 6:134. 10.3389/fped.2018.00134 29868524 PMC5968409

[25] Sun C Kovacs P Guiu-Jurado E. Genetics of obesity in east asians. Front Genet. (2020) 11:575049. 10.3389/fgene.2020.575049 33193685 PMC7606890

[26] Zhang R Wang Z Fei Y Zhou B Zheng S Wang L The difference in nutrient intakes between Chinese and Mediterranean, Japanese and American diets. Nutrients. (2015) 7(6):4661–88. 10.3390/nu7064661 26066014 PMC4488807

[27] Guerrero AD Ponce NA Chung PJ. Obesogenic dietary practices of latino and Asian subgroups of children in California: an analysis of the California health interview survey, 2007-2012. Am J Public Health. (2015) 105(8):e105–12. 10.2105/AJPH.2015.302618 26066936 PMC4504279

[28] Sproesser G Ruby MB Arbit N Akotia CS Alvarenga MDS Bhangaokar R Similar or different? Comparing food cultures with regard to traditional and modern eating across ten countries. Food Res Int. (2022) 157:111106. 10.1016/j.foodres.2022.111106 35761515

[29] Yang J Tani Y Tobias DK Ochi M Fujiwara T. Eating vegetables first at start of meal and food intake among preschool children in Japan. Nutrients. (2020) 12(6):1762. 10.3390/nu12061762 32545520 PMC7353229

[30] Imai S Fukui M Kajiyama S. Effect of eating vegetables before carbohydrates on glucose excursions in patients with type 2 diabetes. J Clin Biochem Nutr. (2014) 54(1):7–11. 10.3164/jcbn.13-67 24426184 PMC3882489

[31] Van Hulst A Paradis G Harnois-Leblanc S Benedetti A Drapeau V Henderson M. Lowering saturated fat and increasing vegetable and fruit intake may increase insulin sensitivity 2 years later in children with a family history of obesity. J Nutr. (2018) 148(11):1838–44. 10.1093/jn/nxy189 30383280 PMC6533243

[32] Ventura AK Worobey J. Early influences on the development of food preferences. Curr Biol. (2013) 23(9):R401–8. 10.1016/j.cub.2013.02.037 23660363

[33] Craigie AM Lake AA Kelly SA Adamson AJ Mathers JC. Tracking of obesity-related behaviours from childhood to adulthood: a systematic review. Maturitas. (2011) 70(3):266–84. 10.1016/j.maturitas.2011.08.005 21920682

[34] Flegal KM Ogden CL Fryar C Afful J Klein R Huang DT. Comparisons of self-reported and measured height and weight, BMI, and obesity prevalence from national surveys: 1999-2016. Obesity (Silver Spring). (2019) 27(10):1711–9. 10.1002/oby.22591 31544344 PMC7289317

[35] Maukonen M Männistö S Tolonen H. A comparison of measured versus self-reported anthropometrics for assessing obesity in adults: a literature review. Scand J Public Health. (2018) 46(5):565–79. 10.1177/1403494818761971 29528773

